# Metabolic and Cognitive Outcomes of Subchronic Once-Daily Intranasal Insulin Administration in Healthy Men

**DOI:** 10.3389/fendo.2018.00663

**Published:** 2018-11-13

**Authors:** Yvonne Ritze, Werner Kern, Eva-Maria Ebner, Serena Jahn, Christian Benedict, Manfred Hallschmid

**Affiliations:** ^1^Department of Medical Psychology and Behavioral Neurobiology, University of Tübingen, Tübingen, Germany; ^2^Endokrinologikum Ulm, Ulm, Germany; ^3^Department of Internal Medicine I, University of Lübeck, Lübeck, Germany; ^4^Department of Neuroscience, Uppsala University, Uppsala, Sweden; ^5^German Center for Diabetes Research (DZD), Neuherberg, Germany; ^6^Institute for Diabetes Research and Metabolic Diseases, Helmholtz Center Munich, University of Tübingen (IDM), Tübingen, Germany

**Keywords:** intranasal insulin, metabolism, body composition, cognitive function, endocrine parameters

## Abstract

Insulin acts in the brain to limit food intake and improve memory function. We have previously shown that 8 weeks of intranasal insulin delivered in four daily doses of 40 IU decrease body weight and enhance word list recall. In the present study, we investigated the effect on body composition, endocrine parameters, and memory performance of 8 weeks of once-daily administration of 160 IU in healthy men. We assumed that intranasal insulin administered before nocturnal sleep, a period of relative metabolic inactivity that moreover benefits memory formation, would be superior to insulin delivery in the morning and placebo administration. After a 2-week baseline period, healthy male normal-weight subjects (mean age, 27.1 ± 0.9 years) received either placebo, 160 IU intranasal insulin in the morning, or 160 IU in the evening (*n* = 12 per group) for 8 consecutive weeks. Throughout the experiment, we measured body weight and body composition as well as circulating concentrations of glucose, insulin, adrenocorticotropin, cortisol, growth hormone, insulin-like growth-factor 1, adiponectin, and leptin. Declarative and procedural memory function was repeatedly assessed by means of, respectively, word list recall and word-stem priming. We found that neither morning nor evening insulin compared to placebo administration induced discernible changes in body weight and body composition. Delayed recall of words showed slight improvements by insulin administration in the evening, and serum cortisol concentrations were reduced after 2 weeks of insulin administration in the morning compared to the other groups. Results indicate that catabolic long-term effects of central nervous insulin delivery necessitate repetitive, presumably pre-meal delivery schedules. The observed memory improvements, although generally weaker than previously found effects, suggest that sleep after intranasal insulin administration may support its beneficial cognitive impact.

## Introduction

Insulin plays an important role in the central nervous control of metabolism and, moreover, improves cognitive function [for reviews, see (1, 2)]. While local insulin production in the cerebral cortex has been suggested based on animal experiments (3), the hormone is not released in large amounts within the CNS and, after pancreatic release, rather reaches the brain via saturable transport mechanisms (4, 5). Insulin receptors are expressed in high densities in the olfactory bulb, the hypothalamus, and the hippocampal formation (6), i.e., structures that are relevant for sensory perception and metabolic regulation as well as the formation of declarative memory contents.

Experiments in animals (7–9) and humans (10, 11) indicate that insulin administered directly to the brain reduces food intake. Many insulin effects on brain function and behavior have been investigated in the human setting by means of the intranasal route of administration, a non-invasive method of delivery that largely bypasses the blood-brain barrier (12, 13). In acute experiments, intranasal administration of 160 IU insulin reduced calorie intake in healthy male participants (10). Young women who received 160 IU intranasal insulin after lunch showed enhanced postprandial satiety and consumed smaller amounts of palatable snacks (11). Daily intranasal insulin administration of 4 × 40 IU (around 30 min before meals and again before going to bed) for 8 weeks decreased body weight and body fat in men but not in women (14). Further evidence for a distinct effect of insulin on food intake-regulatory networks has emerged in neuroimaging studies [(15); see (16, 17) for reviews]. Intranasal insulin has moreover been shown to improve memory function in healthy subjects when delivered acutely (10, 18) or according to the 8-weeks, four-times-per-day schedule described above (19, 20). Patients with mild cognitive impairments and early Alzheimer's disease (AD) likewise benefit from insulin administration [(21, 22) for review see (23)].

Sleep has emerged as an important factor in energy homeostasis and food intake regulation (24). Habitually short sleep is associated with increased body weight (25, 26) and a greater risk of impaired glucose homeostasis (27, 28). Acute sleep deprivation stimulates calorie intake on the subsequent day (29) and leads to a deterioration in glucoregulation (30). In the cognitive domain, the consolidation of memory contents markedly benefits from the brain's offline processing during sleep (31). Neuronal ensembles that encode information during wakefulness are reactivated during subsequent sleep, thereby strengthening respective memory representations (32). We have previously demonstrated that intranasal insulin administration before nocturnal sleep may stabilize memory traces learned in the evening by limiting the interfering influence of encoding new information on the subsequent day (33). Moreover, the acute intranasal administration of 160 IU insulin before nocturnal sleep reduced breakfast intake on the following morning (34). Against this background, we hypothesized that sleep, a period of reduced metabolic activity and largely absent external input, facilitates the emergence of favorable metabolic, and cognitive effects of intranasal insulin. We therefore expected the enhancement of brain insulin signaling during sleep to exceed the effects of repetitive administration of smaller insulin doses throughout the day (14) and, in particular, of insulin administration in the morning. This assumption was tested in young, healthy male subjects who received 160 IU intranasal insulin in the evening or morning, or were treated with placebo for 8 consecutive weeks.

## Methods

### Subjects and design

We included 36 healthy male subjects between 18 and 40 years of age (mean age ± SEM, 27.1 ± 0.9 years) with normal body weight (mean BMI, 23.5 ± 0.3 kg/m^2^). They were all non-smokers and any relevant psychiatric, neurological, cardiovascular, pulmonary, or gastrointestinal disease was excluded before participation by clinical examination and routine laboratory tests. Participants refrained from alcohol, caffeine, or food intake 12 h before each experimental session. They provided written informed consent before the study, which conformed to the Declaration of Helsinki and was approved by the local ethics committee. Experiments were performed in a double-blind manner. Subjects were informed that the study was about the impact of insulin on cortical functions in dependence of body weight and body composition, but were left unaware of the expected memory-improving and catabolic treatment effects. Interviews at the end of the experiment ensured that they did not gain insight into the study purposes.

Subjects were randomly assigned to three groups (each *n* = 12 men) that were comparable regarding age and BMI (*p* > 0.46 for all comparisons). Body fat content averaged across baseline sessions did not significantly differ between the three groups (*p* > 0.10). After 2 weeks of a baseline period of placebo administration in all groups, participants for 8 weeks self-administered, respectively, intranasal insulin after awakening and placebo spray before going to bed (“morning insulin” group; mean age, 27.4 ± 1.1 years, mean BMI, 23.8 ± 0.5 kg/m^2^), placebo spray in the morning and insulin spray before going to bed (“evening insulin” group; mean age, 27.1 ± 1.9 years, mean BMI, 23.3 ± 0.4 kg/m^2^), or placebo spray in the morning and evening (control group; mean age, 26.8 ± 1.8 years, mean BMI, 23.4 ± 0.5 kg/m^2^). Each daily dose was 160 IU insulin (Insulin Actrapid; Novo Nordisk, Mainz, Germany) dissolved in 0.4 ml carrier solution or vehicle administered within four 0.1-ml puffs (two per nostril). Sprays were stored in a refrigerator at 5°C and were replaced every week. Note that before each individual examination, subjects were told to postpone their morning intake routine until after the examination, ensuring that long-term rather than acute effects were assessed. To ensure compliance, subjects kept a diary about their intake routine.

**Figure 1:**
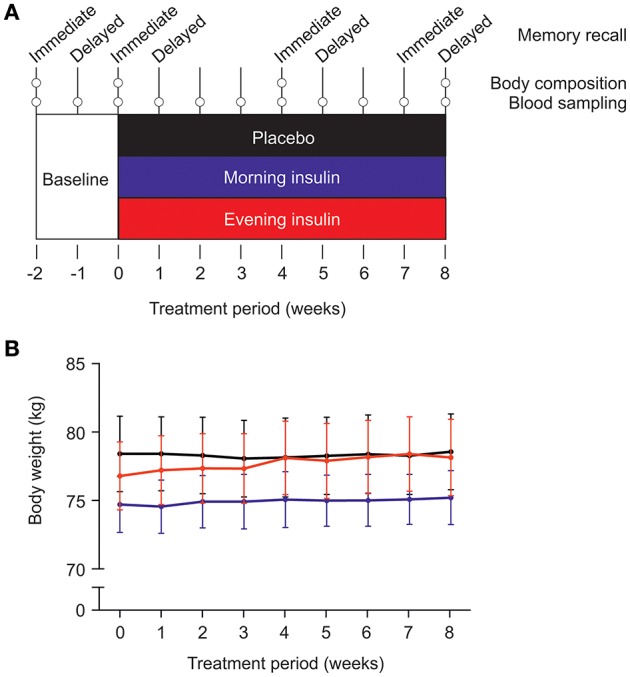
**(A)** Experimental procedure. After a placebo baseline period of 2 weeks, three groups of male subjects (each *N* = 12) were submitted to 8 weeks of intranasal insulin (160 IU) or placebo administration. The “morning insulin” group self-administered insulin after awakening (or after the weekly examination) and placebo spray before going to bed; the “evening insulin” group self-administered placebo spray in the morning and insulin spray before going to bed; the control group received placebo in the morning and evening. Metabolic and cognitive assessments took place as depicted; for methodological details, see text. **(B)** Average body weight (±SEM) in the three groups during insulin intervention or placebo treatment.

Four major test sessions (scheduled between 07:00 and 09:00 h) were conducted, i.e., at the start of the baseline period, after 2 weeks of baseline placebo administration, and after 4 and 8 weeks of insulin or placebo treatment (see Figure 1A for an overview over the experimental design). Subjects were weighed (as well as on a weekly basis, see below) and their body composition was measured by standard bioelectrical impedance analysis (frequencies of 1, 5, 50, and 100 Hz; BIA 2000-M; Data Input, Frankfurt, Germany) indicating body fat, total body water, intracellular water, extracellular water, lean body mass, and body cell mass (Eurobody software; Data Input). Waist circumference was also measured, and subjects completed a questionnaire on their eating behavior [FEV; (35)]. Participants rated their hunger, thirst, and tiredness on 10-point scales in the beginning and at the end of the session, yielding difference values indicating the current gradient of these parameters. In order to control for possible side effects, we also monitored blood pressure and heart rate, as well as routine laboratory measurements (serum electrolytes; creatinine; HDL, LDL, and total cholesterol; triglycerides; data not reported).

### Psychological assessments

#### Word list

In this test of declarative memory, a list of 30 words was presented and recalled immediately as well as after a 1-week delay. (Note that the final assessment of immediate recall took place after 7 weeks of the insulin intervention in order to accommodate the final assessment of delayed recall after 8 weeks of treatment). The words belonged to three semantic categories, neutral (e.g., “wind,” “moss”), food-related (e.g., “pineapple,” “cheese”), and emotional (e.g., “joy,” “cock”), and were presented orally at a rate of one word/s. Subsequently, subjects were told to remain silent for a break of 3 min and to keep the presented words in mind. For immediate recall, subjects wrote down all words they remembered within 90 s. For delayed recall, ~1 week later, subjects again had to write down all words they still remembered from this list (19, 20, 36). Because the respective morning insulin or placebo administrations took place after the test session, the study design did not allow for testing acute insulin effects on immediate or delayed recall. In short post-treatment interviews none of the subjects stated to have learned or thought about the word list within the week before delayed recall, excluding major interfering influences of rehearsal effects.

#### Word-stem priming

Non-declarative memory was tested with a word-stem priming task based on a learning word list and a test list of two-letter word-stems. First, subjects rated the nouns of the learning word list according to their sound on a 5-point scale (from 1 = unpleasant to 5 = pleasant). This task was considered to induce implicit learning. Thereafter the subjects received the test list containing 52 two-letter word-stems (e.g. “ho” derived from “hotel”). Twenty-six word-stems of this list were derived from the (rated) learning list, whereas the other 26 word-stems were taken from a pool of new words not presented to the subject (new list). Subjects were instructed to complete the word-stems to the first noun that came to their mind. The difference between the number of word-stems completed to nouns from the learning list and the number of words accidentally completed to nouns of the new list was considered a measure of implicit memory (19, 37). Parallel versions of the word list and the word-stem priming task were used for each subject in the four test sessions, and their order was balanced across subjects.

#### Mood

During each major test session, subjects filled in an adjective check list designed to assess current mood and feelings of activation [Eigenschaftswörterliste EWL-N; (38)]. The adjective check list consists of a total of 161 adjectives grouped into 15 dimensions, i.e., activation, concentration, deactivation, tiredness, numbness, extraversion, introversion, self-assuredness, mood, excitation, sensitivity, anger, anxiousness, depression, and dreaminess. For each adjective, the subject had to indicate whether or not it reflected aspects of his current state of mood. For each dimension, the number of adjectives marked by the subject was counted and transformed to percentages of the respective achievable maximum value.

### Blood parameters

Weekly, around 08:00 h, subjects were weighed and blood samples were collected. Immediately after blood drawing, blood samples were centrifuged and plasma and serum were stored at −20°C. Concentrations of leptin, insulin, adrenocorticotropin, cortisol, and adiponectin were assessed using standard radioimmunoassays (Human Leptin RIA KIT, Linco Research, St. Charles, MO; Pharmacia Insulin RIA100, Pharmacia Pharmacia & Upjohn, Uppsala, Sweden; Lumitest ACTH, Brahms Diagnostica, Hennigsdorf, Germany; Cortisol-RIA, DPC Biermann GmbH, Bad Nauheim, Germany; HADP-61HK adiponectin kit, Linco Research, St. Charles, MO). Serum concentrations of growth hormone (Immulite, DPC, Los Angeles, CA, USA) and insulin-like growth factor (IGF-I; Active IGF-I, Diagnostics Systems Laboratories, Inc., Sinsheim, Germany) were measured by ELISA. Plasma glucose was measured spectrophotometrically with the Hexokinase/G-6-PDH assay (Aeroset, Abbott, Wiesbaden, Germany). Intervals between weekly sessions were 7 days but adjusted to minor extents in order to accommodate individual schedules of the participants.

### Statistical analyses

Statistical analyses were based on analyses of variance (ANOVA) with the between-subject factor “group” and the within-subject factor “time.” Analyses of psychological tasks included baseline values as covariates to take into account interindividual variations. Also, individual delays between sessions (expressed as number of days) were introduced as covariates into the analyses of delayed word list recall and word-stem priming to adjust for variations in retrieval intervals. In order to obtain a measure of declarative memory decay, immediate recall performance on the word list task was subtracted from delayed recall values. Student's *t*-tests for independent samples were used for pairwise *post-hoc* comparisons between groups. Values are expressed as means ± SEM and a *p*-value < 0.05 was considered significant.

## Results

### Body weight, body composition, and eating-related assessments

Body weight of the three groups, morning insulin, evening insulin, and control, generally increased during the treatment period, i.e., between the last baseline examination and the session after 8 weeks of insulin or placebo administration [*F*_(4, 144)_ = 2.41, *p* = 0.046 for *Time*; Figure 1B]. There were no differences between groups [*F*_(8, 144)_ = 1.26, *p* = 0.26 for *Group* × *Time*; *F*_(2, 33)_ = 0.16, *p* = 0.86 for *Group*], and neither any differences between morning and evening insulin administration (*p* > 0.39). BMI-values mirrored this pattern [*F*_(4, 143)_ = 2.32, *p* = 0.055 for *Time* and *p* > 0.31 for treatment-related comparisons]. Body fat content likewise displayed a general trend toward increased values between baseline and final examination [*F*_(2, 44)_ = 2.5, *p* = 0.09] which, however, did not depend on insulin treatment (*p* > 0.13). In the same time period, fat-free mass remained unchanged (*p* > 0.77) and was likewise not altered by insulin treatment (all *p* > 0.10) as were body cell mass, body water and intracellular water (all *p* > 0.10). Even before the insulin intervention, extracellular water appeared generally decreased in the group receiving insulin in the morning compared to the evening insulin and the control group [*F*_(2, 33)_ = 4.65, *p* = 0.017 for *Group*], with no time-dependent changes to this pattern (all *p* > 0.31). See Table 1 for a summary of body composition measures. Waist circumference did not change over time nor in dependence of insulin treatment (*p* > 0.30).

**Table 1:** Body composition.

	**Placebo**	**Morning insulin**	**Evening insulin**
**BASELINE**			
Body fat (kg)	11.38 ± 1.34	14.48 ± 1.05	12.05 ± 1.43
Fat free mass (kg)	67.08 ± 1.82	61.76 ± 1.58	65.28 ± 1.49
Total body water (kg)	49.12 ± 1.34	45.22 ± 1.15	47.78 ± 1.09
Intracellular water (kg)	28.98 ± 0.86	27.08 ± 0.74	28.31 ± 0.70
Extracellular water (kg)	20.14 ± 0.51	18.14 ± 0.44^*^	19.48 ± 0.43
Body cell mass (kg)	37.95 ± 1.14	35.14 ± 0.96	37.85 ± 0.87
**4 WEEKS OF TREATMENT**			
Body fat (kg)	11.66 ± 1.25	14.65 ± 1.12	11.53 ± 1.38
Fat free mass (kg)	66.52 ± 2.17	61.77 ± 1.66	66.62 ± 1.73
Total body water (kg)	48.71 ± 1.59	45.23 ± 1.21	48.76 ± 1.27
Intracellular water (kg)	28.78 ± 0.97	27.16 ± 0.79	28.78 ± 0.82
Extracellular water (kg)	19.93 ± 0.64	18.07 ± 0.45^*^	19.98 ± 0.49
Body cell mass (kg)	37.61 ± 1.31	35.28 ± 1.05	38.15 ± 0.92
**8 WEEKS OF TREATMENT**			
Body fat (kg)	11.65 ± 1.17	15.21 ± 1.13	12.48 ± 1.49
Fat free mass (kg)	66.94 ± 2.18	61.69 ± 1.61	65.71 ± 1.66
Total body water (kg)	49.02 ± 1.59	45.17 ± 1.18	48.10 ± 1.21
Intracellular water (kg)	28.94 ± 0.96	27.17 ± 0.76	28.63 ± 0.81
Extracellular water (kg)	20.08 ± 0.66	18.00 ± 0.45^*^	19.48 ± 0.44
Body cell mass (kg)	37.87 ± 1.21	35.35 ± 1.07	37.94 ± 1.02

Results are mean ± SEM. N = 12 per group.

* *p < 0.05 for comparisons between the morning insulin and placebo/evening insulin groups*.

Hunger ratings remained constant across the experimental period and were not modulated by treatment (all *p* > 0.32). Values of the subscales “hunger” and “suggestibility” of the eating behavior questionnaire were likewise independent of time and treatment (all *p* > 0.09). “Cognitive control” according to this questionnaire was generally more strongly expressed in the morning insulin group [6.1 ± 0.8, averaged across the results obtained at the end of the baseline and after 4 and 8 weeks of treatment; *F*_(2, 33)_ = 3.90, *p* = 0.03 for *Group*] than in the evening insulin (3.9 ± 1.0; *p* = 0.09) and the control group (2.8 ± 0.7; *p* = 0.008), but did not change during the intervention (*p* > 0.39 for *Time* × *Group* and *Time*). Thirst ratings were stable and unrelated to the intervention (*p* > 0.20 for all comparisons). The analysis of tiredness ratings indicated a significant interaction between the factors *Group* and *Time* [*F*_(4, 61)_ = 3.77, *p* = 0.009; *p* > 0.41 for the factors *per se*] that was due to an effect of insulin administration in the morning [*F*_(2, 33)_ = 3.58, *p* < 0.05; *p* > 0.19 for comparisons between evening insulin and placebo]. Thus, after 4 weeks of morning insulin administration, rated tiredness showed a steeper decline during the experimental session (−1.1 ± 0.3) than both after evening insulin (−0.2 ± 0.2; *p* = 0.01) and placebo (−0.08 ± 0.4; *p* = 0.04).

Control assessments of hemodynamic parameters did not indicate robust treatment effects or changes across time. Averaged across the experimental period, diastolic blood pressure reached values of 71.59 ± 1.63 mmHg (morning insulin), 72.11 ± 2.01 mmHg (evening insulin), and 72.30 ± 2.16 mmHg (placebo; *p* > 0.07 for all comparisons). Systolic blood pressure was 130.23 ± 2.02 mmHg (morning insulin), 129.10 ± 2.83 (evening insulin), and 127.88 ± 2.36 mmHg (placebo; *p* > 0.06). Heart rate averaged 63.77 ± 1.76 bpm (morning insulin), 67.60 ± 2.93 bpm (evening insulin), and 69.11 ± 1.99 bpm (placebo; *p* > 0.24).

### Memory tasks and mood

#### Word list

Immediate recall of words was generally well comparable at baseline (*p* > 0.16 for all overall comparisons), although for emotional words, the performance level in the morning insulin group tended to be below that of the placebo group (Table 2; note that post-baseline outcomes are baseline-adjusted). Insulin treatment did not have a systematic effect on the immediate recall of words. Delayed recall of words (assessed 1 week after encoding) appeared to benefit from insulin administration in the evening. For the sum of all words recalled after 5 weeks of treatment, the ANCOVA factor *Group* displayed a trend [*F*_(2, 30)_ = 2.73, *p* = 0.08], and participants of the evening insulin compared to the morning insulin group performed better on the recall of neutral as well as of all words, with the placebo group in-between. There were also signs of improvements in the delayed recall of emotional words after 5 weeks of insulin administration in the evening vs. morning and placebo (Table 2).

**Table 2:** Immediate and delayed word list recall.

	**Placebo**	**Morning insulin**	**Evening insulin**
**IMMEDIATE RECALL**			
**Baseline**			
Food-related	3.79 ± 0.37	3.25 ± 0.37	3.29 ± 0.32
Emotional	4.42 ± 0.34	3.54 ± 0.265^a^	4.17 ± 0.37
Neutral	3.50 ± 0.40	3.08 ± 0.34	3.46 ± 0.34
All words	11.71 ± 0.89	9.88 ± 0.83	10.92 ± 0.71
**4 weeks of treatment**			
Food-related	3.73 ± 0.35	3.23 ± 0.34	3.37 ± 0.34
Emotional	4.35 ± 0.46	4.64 ± 0.47	4.34 ± 0.45
Neutral	4.75 ± 0.49	3.48 ± 0.495^a^	4.27 ± 0.49
All words	12.51 ± 0.89	11.80 ± 0.90	11.85 ± 0.88
**7 weeks of treatment**			
Food-related	3.83 ± 0.45	3.76 ± 0.45	4.07 ± 0.44
Emotional	4.63 ± 0.47	4.36 ± 0.47	3.93 ± 0.46
Neutral	4.15 ± 0.55	3.50 ± 0.55	4.26 ± 0.55
All words	12.40 ± 1.10	11.83 ± 1.10	12.27 ± 1.08
**DELAYED RECALL**			
**1 week of treatment**			
Food-related	1.84 ± 0.41	0.93 ± 0.41	1.06 ± 0.41
Emotional	1.44 ± 0.46	1.39 ± 0.48	2.25 ± 0.46
Neutral	1.47 ± 0.30	1.47 ± 0.30	1.15 ± 0.31
All words	4.60 ± 0.82	3.84 ± 0.81	4.56 ± 0.82
**5 weeks of treatment**			
Food-related	1.26 ± 0.35	0.48 ± 0.35	1.10 ± 0.36
Emotional	1.19 ± 0.37	1.16 ± 0.37	2.15 ± 0.375^b^
Neutral	1.69 ± 0.48	0.81 ± 0.48	2.25 ± 0.48^*^
All words5^c^	3.96 ± 0.91	2.56 ± 0.90	5.57 ± 0.915^*^
**8 weeks of treatment**			
Food-related	1.19 ± 0.33	0.72 ± 0.36	1.08 ± 0.33
Emotional	0.98 ± 0.31	1.31 ± 0.31	1.63 ± 0.32
Neutral	0.96 ± 0.41	1.05 ± 0.41	1.02 ± 0.42
All words	3.03 ± 0.78	3.13 ± 0.78	3.85 ± 0.80

Results are mean ± SEM. N = 12 per group.

a p < 0.10 for comparison between the placebo and the morning insulin group.

b p < 0.10 for comparison between the evening insulin and the placebo/morning insulin groups.

c p < 0.10 for ANCOVA factor group.

* *p < 0.05 for comparisons between the evening and morning insulin groups*.

**Figure 2:**
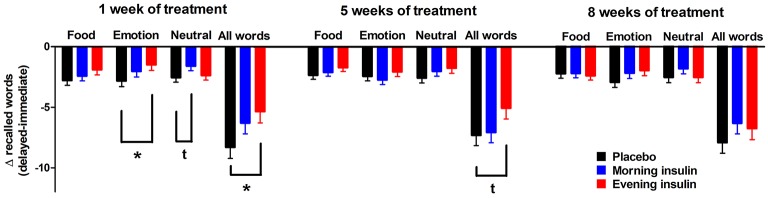
Memory decay between immediate and delayed word recall. Differences (±SEM) between the numbers of words (food-related, emotional, neutral, and all words) from the word list recalled in the delayed and the immediate sessions, which took place roughly 1 week apart. Values were adjusted by ANCOVA for baseline differences and the individual temporal delays between immediate and delayed recall. *N* = 12 per group; ^*^p < 0.05, ^t^*p* < 0.10 for comparisons between respective groups.

We also analyzed the differences between immediate and delayed word list recall to obtain a measure of forgetting and found that memory decay was less pronounced in the evening insulin than the placebo group, with morning insulin in-between, in roughly half of sessions and categories combined (Figure 2). This pattern was corroborated on a tendency level for the sum of words recalled after 1 week of administration [*F*_(2, 30)_ = 2.61, *p* = 0.09], when it seemed particularly salient for emotional words, but was likewise visible after 5 weeks and, on a descriptive level, also 8 weeks of treatment. Morning insulin administration appeared to curb the decay of memory for neutral words assessed after 1 week of treatment.

In exploratory *post-hoc* analyses, we investigated the potential modulatory impact on word list recall of peripheral insulin sensitivity as reflected by homeostatic model assessment insulin resistance (HOMA-IR) calculated from fasting serum insulin and plasma glucose values obtained at the start of the baseline period, and introduced in analyses covering the whole treatment period as an additional between-subjects factor (low/high) derived from group-specific median splits. We did not find indicators for interactions between insulin effects and peripheral insulin sensitivity with regard to immediate word list recall (all *p* > 0.30 for *Group* × *HOMA*). Delayed total word list recall, in contrast, particularly benefited from (evening) insulin compared to placebo administration in subjects with relatively high peripheral insulin sensitivity [*F*_(2, 25)_ = 3.42, *p* = 0.049 for *Group* × *HOMA*; estimated marginal means, 5.49 ± 0.88, 4.45 ± 0.87, and 3.22 ± 0.92 words in the evening/morning insulin and placebo groups, vs. 4.28 ± 0.79, 1.80 ± 0.69, and 4.14 ± 0.76 words in the respective groups with low insulin sensitivity, *F*_(2, 25)_ = 3.12, *p* = 0.062 for *Group*]. Signs of a comparable pattern were obtained in sub-analyses of emotional words [*F*_(2, 25)_ = 2.91, *p* = 0.073 for *Group* × *HOMA*; 2.48 ± 0.31, 1.77 ± 0.31, and 0.69 ± 0.34 words in the evening/morning insulin and placebo groups with high insulin sensitivity, vs. 1.80 ± 0.40, 0.96 ± 0.37, and 1.30 ± 0.38 words in the respective groups with low insulin sensitivity, *F*_(2, 25)_ = 3.76, *p* = 0.037 for *Group*]. The impact of insulin on memory decay did not interact with HOMA-derived insulin sensitivity (all *p* > 0.25).

#### Word-stem priming

Performance on the word-stem priming task remained completely unaffected by insulin treatment both during immediate (*p* > 0.52 for all overall comparisons) and delayed testing (*p* > 0.46; see Table 3 for detailed results).

**Table 3:** Results of the word-stem priming task.

	**Placebo**	**Morning insulin**	**Evening insulin**
**IMMEDIATE RECALL**			
Baseline	4.15 ± 0.67	3.47 ± 0.67	3.22 ± 0.67
4 weeks of treatment	3.75 ± 0.65	3.41 ± 0.65	3.34 ± 0.65
7 weeks of treatment	4.46 ± 0.82	4.94 ± 0.82	4.10 ± 0.82
**DELAYED RECALL**			
1 week of treatment	0.82 ± 0.42	0.81 ± 0.42	0.62 ± 0.42
5 weeks of treatment	0.37 ± 0.46	0.59 ± 0.45	0.96 ± 0.46
8 weeks of treatment	1.05 ± 0.46	1.80 ± 0.46	1.15 ± 0.47

*Results are mean ± SEM. N = 12 per group*.

#### Mood

Results of the adjective scale provided to our participants to self-rate current mood on 15 dimensions indicated that self-rated concentration was enhanced in the participants of the insulin groups compared to those of the placebo group after 4 and 8 weeks of treatment [*F*_(2, 31)_ = 5.54, *p* = 0.009 for *Group*; *p* > 0.12 for *Time* and interaction], reaching mean values of 72.88 ± 5.80% (evening insulin), 73.39 ± 5.80% (morning insulin), and 48.76 ± 6.06% in the placebo group. All other scores remained unchanged, i.e., activation (*p* > 0.15 for the factors *Group, Time*, and respective interaction), deactivation (*p* > 0.12), tiredness (*p* > 0.13), numbness (*p* > 0.63), extraversion (*p* > 0.20), introversion (*p* > 0.64), self-assuredness (*p* > 0.17), mood (*p* > 0.57), excitation (*p* > 0.21), sensitivity (*p* > 0.21), anger [*F*_(1, 31)_ = 3.73, *p* > 0.06 for *Time*; *p* > 0.31 for *Group* and interaction], anxiousness (*p* > 0.28), depression (*p* > 0.91), and dreaminess (*p* > 0.32).

### Blood parameters

**Figure 3:**
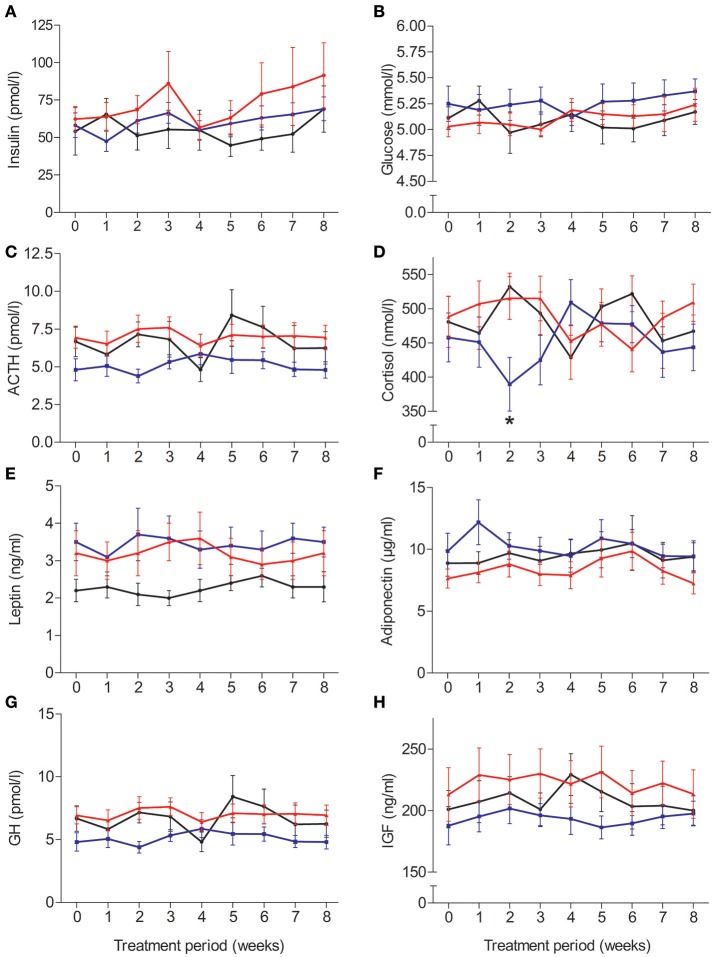
Average (±SEM) serum or plasma concentrations of **(A)** insulin, **(B)** glucose, **(C)** adrenocorticotropin, **(D)** cortisol, **(E)** leptin, **(F)** adiponectin, **(G)** growth hormone, and **(H)** insulin-like growth factor. *N* = 12; ^*^p < 0.05 for comparisons between the morning and evening insulin/placebo groups.

Circulating concentrations of glucose and endocrine parameters, except for serum cortisol, remained unaffected by insulin treatment (Figure 3). No group differences emerged for serum insulin and plasma glucose (*p* > 0.16 for *Group* and *Group* × *Time*), which also remained stable during the experimental period (*p* > 0.10). While plasma adrenocorticotropin was not altered by any of the insulin interventions (*p* > 0.14) and temporal fluctuations failed to reach significance [*F*_(6, 188)_ = 2.08, *p* = 0.06], serum cortisol concentrations were suppressed in the morning insulin compared to both other groups after 2 weeks of administration [Figure 3D; *F*_(15, 242)_ = 1.95, *p* = 0.02 for *Group* × *Time*; *p* > 0.42 for *Group* and *Time*]. Serum leptin concentrations remained unchanged (all *p* > 0.10); plasma adiponectin concentrations did not respond to treatment (all *p* > 0.49) but appeared to increase once a month independent of treatment, with a respective trough at the end of experiments [Figure 3F; *F*_(5, 177)_ = 2.36, *p* = 0.04]. There were no robust treatment effects on serum concentrations of growth hormone (all *p* > 0.09) and IGF-1 (*p* > 0.41, *p* > 0.06 for *Time*).

## Discussion

Building on our previous studies in which we administered four daily doses of 40 IU intranasal insulin (14, 19), here we investigated the metabolic and cognitive outcomes of 8-week once-daily administration of 160 IU insulin in healthy men. We expected to find superior effects of insulin administration in the evening compared to delivery in the morning and placebo, assuming that enhanced brain insulin signaling and sleep would interact to improve metabolic control and cognitive function. Neither the evening nor the morning schedule of insulin delivery exerted traceable effects on body weight; signs of improved declarative memory consolidation during evening compared to morning insulin and placebo administration were modest and restricted to the first weeks of treatment. Insulin treatment however reduced circulating cortisol concentrations and exerted stimulating psychobehavioral effects, demonstrating the principal efficacy of our intervention.

Against the background of a slight general increase in body weight and body fat content across the experimental period, we did not detect robust effects on body weight and body composition of intranasal insulin delivered in the morning or evening, which stands in contrast to our previous observation of an insulin-induced loss of around 1.4 kg body fat in healthy men who received the peptide four times a day, i.e., before main meal intake and before going to bed (14). Those subjects also displayed signs of insulin-induced reductions in hunger that were absent in the participants of the current study. Independent of insulin treatment, the subjects of the morning insulin group had higher levels of extracellular water than those of the other groups, so that subtle interactions between central insulin signaling and water homeostasis cannot be ruled out (14, 39). However, the participants who received insulin in the evening neither showed insulin-induced changes in body composition. This result is particularly puzzling because in previous experiments, the intranasal administration of 160 IU insulin to healthy men before bedtime led to an acute reduction of breakfast intake by 175 kcal (34). Since body weight in the evening insulin group was not affected even in the first weeks of treatment, this suggests that central nervous insulin administration before sleep might lose its catabolic impact rather quickly. Alternatively, counteracting mechanisms like centrally mediated increases in lipogenesis (40) might set in which, however, could not be investigated in the present experiments. It should also be noted that animal experiments have not unanimously shown hypophagic effects of central insulin delivery (41, 42). Taken together and with a view to potential clinical applications, the current data indicate that the effects of intranasal insulin on body weight and body fat are clearly stronger when smaller individual doses (e.g., 40 IU) are delivered before meal or snack intake (10, 11, 14).

After 2 weeks of treatment onset, insulin administration in the morning compared to the evening, and to placebo, reduced cortisol concentrations. This finding is generally in line with the (albeit more pronounced and persistent) insulin-induced suppression of circulating cortisol in obese men receiving 4 × 40 IU insulin/day (43) and with results found at the end of the 8-week treatment period in normal-weight men (19). Dampening effects of central insulin on hypothalamo-pituitary-adrenal (HPA) axis activity may be mediated by enhanced corticosteroid feedback processing in the hippocampus (44), which is assumed to exert inhibiting control over the HPA system via projections to the hypothalamus (45). The weak and transient nature of the effect obtained in the present experiments, however, again underlines that once-daily insulin administration paradigms may not be optimally suited to induce neuroendocrine effects. Previously, acute cortisol-lowering effects of evening intranasal insulin administration were found in aging but not in young healthy subjects (46). The absence of robust hemodynamic effects corroborates our previous finding that central insulin administration raises blood pressure acutely, but not after long-term delivery (47).

In the cognitive domain, we merely detected subtle insulin effects that suggest greater efficacy of insulin delivery in the evening compared to the morning but do not identify once-daily administration as a very suitable paradigm to boost memory performance in healthy humans. Memory decay between immediate and delayed word recall appeared mitigated within 1 week and delayed recall appeared to be generally enhanced after 5 weeks of evening insulin administration. These mildly beneficial effects of pre-sleep insulin delivery connect to our previous investigation into the acute impact of intranasal insulin given before bedtime on sleep-associated memory formation (33). In that study, insulin did not directly improve the consolidation of declarative memory contents, but impaired the acquisition of new, interfering contents learned on the subsequent day, suggesting that the peptide inhibits processes of active forgetting during sleep (48). Such changes may support word list recall, but the present findings indicate that respective insulin-induced improvements wane with prolonged treatment. In contrast, memory-enhancing effects of 4 × 40 IU/day insulin administration emerged only after 8 weeks of treatment [memory recall after 5 weeks was not tested in those studies; (19, 20)], but were stronger than those found in the present study. Thus, the temporal dynamics of insulin's memory effect in dependence of administration schedules is in need of further investigation, as are the underlying mechanisms that are assumed to involve insulin receptors located in the hippocampus and connected limbic brain structures (6) in as much as down-regulating hippocampal insulin receptor function impairs long term potentiation and spatial memory (49). Improved self-rated concentration and reduced tiredness due to insulin delivery might moreover have enhanced cognitive function. In all three groups, delayed word recall performance slightly declined across the repeated presentations of the task. While parallel versions were used in balanced order, this pattern was most probably due to proactive interferences from intrusions, i.e., falsely recalled words from previous lists (19). Our observation that the insulin effect on declarative memory was somewhat more pronounced in subjects with relatively high as compared to those with low peripheral insulin sensitivity is in accordance with neuroimaging studies that suggest parallel decreases in central nervous and peripheral insulin sensitivity (50, 51) but should be followed up in larger cohorts.

In line with our previous experiments (19, 20), the immediate recall of words and non-declarative memory function, as assessed by the word-stem priming task, were not affected by insulin. However, the morning insulin compared to the evening insulin and the control groups displayed signs of generally weaker immediate recall performance, which limits respective conclusions. While group sizes were generally comparable to those of previous studies (19–21, 52), larger samples might be needed to corroborate and expand the present findings. They should also include female subjects, although previous experiments suggest that the cognitive impact of intranasal insulin differs between men and women after acute (10) but not long-term administration (19).

In sum, our finding that once-daily intranasal administration of 160 IU insulin does not affect body weight regulation and only slightly improves declarative memory function when scheduled in the evening may be of particular relevance for potential clinical applications in the metabolic as well as cognitive domain (17, 53). The results imply that subchronic once-daily administration of high insulin doses is inferior rather than superior to treatment regimens spread across the day. Considering that obese men treated with 4 × 40 IU/d of intranasal insulin show memory improvements but no change in body weight (43), they also support the tentative assumption that the cognitive impact of intranasal insulin is generally more robust than its metabolic outcomes. Central nervous insulin delivery has long been proposed as a promising intervention to alleviate cognitive impairments for example in patients with AD (22, 54). In this context, the timing of insulin administration certainly deserves a closer look, not least when taking into account interactions with sleep-associated processes.

## Author contributions

WK, CB, and MH designed the study. E-ME and SJ enrolled subjects, performed experiments and contributed to data analyses. YR and MH analyzed and interpreted the data and wrote the manuscript.

### Conflict of interest statement

The authors declare that the research was conducted in the absence of any commercial or financial relationships that could be construed as a potential conflict of interest.

## References

[1] Lee SH Zabolotny JM Huang H Lee H Kim YB. Insulin in the nervous system and the mind: functions in metabolism, memory, and mood. Mol Metab. (2016) 5:589–601. 10.1016/j.molmet.2016.06.011 27656397 PMC5021669

[2] Benedict C Grillo CA. Insulin resistance as a therapeutic target in the treatment of Alzheimer's disease: a state-of-the-art review. Front Neurosci. (2018) 12:215. 10.3389/fnins.2018.00215 29743868 PMC5932355

[3] Molnar G Farago N Kocsis AK Rozsa M Lovas S Boldog E. GABAergic neurogliaform cells represent local sources of insulin in the cerebral cortex. J Neurosci. (2014) 34:1133–7. 10.1523/JNEUROSCI.4082-13.2014 24453306 PMC6705313

[4] Baura GD Foster DM Porte D Jr Kahn SE Bergman RN Cobelli C. Saturable transport of insulin from plasma into the central nervous system of dogs *in vivo*. a mechanism for regulated insulin delivery to the brain. J Clin Invest. (1993) 92:1824–30. 10.1172/JCI116773 8408635 PMC288346

[5] Gray SM Meijer RI Barrett EJ. Insulin regulates brain function, but how does it get there? Diabetes (2014) 63:3992–7. 10.2337/db14-0340 25414013 PMC4237995

[6] Unger JW Livingston JN Moss AM. Insulin receptors in the central nervous system: localization, signalling mechanisms and functional aspects. Prog Neurobiol. (1991) 36:343–62. 10.1016/0301-0082(91)90015-S 1887067

[7] Woods SC Lotter EC Mckay LD Porte D Jr. Chronic intracerebroventricular infusion of insulin reduces food intake and body weight of baboons. Nature (1979) 282:503–5. 10.1038/282503a0 116135

[8] McGowan MK Andrews KM Grossman SP. Chronic intrahypothalamic infusions of insulin or insulin antibodies alter body weight and food intake in the rat. Physiol Behav. (1992) 51:753–66. 10.1016/0031-9384(92)90112-F 1317588

[9] Air EL Benoit SC Blake Smith KA Clegg DJ Woods SC. Acute third ventricular administration of insulin decreases food intake in two paradigms. Pharmacol Biochem Behav. (2002) 72:423–9. 10.1016/S0091-3057(01)00780-8 11900815

[10] Benedict C Kern W Schultes B Born J Hallschmid M. Differential sensitivity of men and women to anorexigenic and memory-improving effects of intranasal insulin. J Clin Endocrinol Metab. (2008) 93:1339–44. 10.1210/jc.2007-2606 18230654

[11] Hallschmid M Higgs S Thienel M Ott V Lehnert H. Postprandial administration of intranasal insulin intensifies satiety and reduces intake of palatable snacks in women. Diabetes (2012) 61:782–9. 10.2337/db11-1390 22344561 PMC3314365

[12] Born J Lange T Kern W Mcgregor GP Bickel U Fehm HL. Sniffing neuropeptides: a transnasal approach to the human brain. Nat Neurosci. (2002) 5:514–6. 10.1038/nn0602-849 11992114

[13] Dhuria SV Hanson LR Frey WH III. Intranasal delivery to the central nervous system: mechanisms and experimental considerations. J Pharm Sci. (2010) 99:1654–73. 10.1002/jps.21924 19877171

[14] Hallschmid M Benedict C Schultes B Fehm HL Born J Kern W Intranasal insulin reduces body fat in men but not in women. Diabetes (2004) 53:3024–9. 10.2337/diabetes.53.11.3024 15504987

[15] Tiedemann LJ Schmid SM Hettel J Giesen K Francke P Büchel C. Central insulin modulates food valuation via mesolimbic pathways. Nat Commun. (2017) 8:16052. 10.1038/ncomms16052 28719580 PMC5520049

[16] Heni M Kullmann S Preissl H Fritsche A Haring HU. Impaired insulin action in the human brain: causes and metabolic consequences. Nat Rev Endocrinol. (2015) 11:701–11. 10.1038/nrendo.2015.173 26460339

[17] Kullmann S Heni M Hallschmid M Fritsche A Preissl H Haring HU. Brain insulin resistance at the crossroads of metabolic and cognitive disorders in humans. Physiol Rev. (2016) 96:1169–209. 10.1152/physrev.00032.2015 27489306

[18] Brunner YF Kofoet A Benedict C Freiherr J. Central insulin administration improves odor-cued reactivation of spatial memory in young men. J Clin Endocrinol Metab. (2015) 100:212–9. 10.1210/jc.2014-3018 25337926

[19] Benedic C Hallschmid M Hatke A Schultes B Fehm HL Born J Intranasal insulin improves memory in humans. Psychoneuroendocrinology (2004) 29:1326–34. 10.1016/j.psyneuen.2004.04.003 15288712

[20] Benedict C Hallschmid M Schmitz K Schultes B Ratter F Fehm HL. Intranasal insulin improves memory in humans: superiority of insulin aspart. Neuropsychopharmacology (2007) 32:239–43. 10.1038/sj.npp.1301193 16936707

[21] Reger MA Watson GS Green PS Wilkinson CW Baker LD Cholerton B. Intranasal insulin improves cognition and modulates beta-amyloid in early AD. Neurology (2008) 70:440–8. 10.1212/01.WNL.0000265401.62434.36 17942819

[22] Craft S Baker LD Montine TJ Minoshima S Watson GS Claxton A. Intranasal insulin therapy for Alzheimer disease and amnestic mild cognitive impairment: a pilot clinical trial. Arch Neurol. (2012) 69:29–38. 10.1001/archneurol.2011.233 21911655 PMC3260944

[23] de Felice FG. Alzheimer's disease and insulin resistance: translating basic science into clinical applications. J Clin Invest. (2013) 123:531–9. 10.1172/JCI64595 23485579 PMC3561831

[24] St-Onge MP. The role of sleep duration in the regulation of energy balance: effects on energy intakes and expenditure. J Clin Sleep Med. (2013) 9:73–80. 10.5664/jcsm.2348 23319909 PMC3525993

[25] Magee L Hale L. Longitudinal associations between sleep duration and subsequent weight gain: a systematic review. Sleep Med Rev. (2012) 16:231–41. 10.1016/j.smrv.2011.05.005 21784678 PMC3202683

[26] Vgontzas AN Fernandez-Mendoza J Miksiewicz T Kritikou I Shaffer ML Liao D. Unveiling the longitudinal association between short sleep duration and the incidence of obesity: the penn state cohort. Int J Obes (Lond). (2014) 38:825–32. 10.1038/ijo.2013.172 24100421 PMC3954466

[27] Gangwisch JE Heymsfield SB Boden-Albala B Buijs RM Kreier F Pickering TG. Sleep duration as a risk factor for diabetes incidence in a large U.S. sample. Sleep (2007) 30:1667–73. 10.1093/sleep/30.12.1667 18246976 PMC2276127

[28] Cappuccio FP D'Elia L Strazzullo P Miller MA. Quantity and quality of sleep and incidence of type 2 diabetes: a systematic review and meta-analysis. Diabetes Care (2010) 33:414–20. 10.2337/dc09-1124 19910503 PMC2809295

[29] Brondel L Romer MA Nougues PM Touyarou P Davenne D. Acute partial sleep deprivation increases food intake in healthy men. Am J Clin Nutr. (2010) 91:1550–9. 10.3945/ajcn.2009.28523 20357041

[30] Schmid SM Hallschmid M Jauch-Chara K Wilms B Lehnert H Born J. Disturbed glucoregulatory response to food intake after moderate sleep restriction. Sleep (2011) 34:371–7. 10.1093/sleep/34.3.371 21358855 PMC3041714

[31] Feld GB Born J. Sculpting memory during sleep: concurrent consolidation and forgetting. Curr Opin Neurobiol. (2017) 44:20–7. 10.1016/j.conb.2017.02.012 28278432

[32] Diekelmann S Born J. The memory function of sleep. Nat Rev Neurosci. (2010) 11:114–26. 10.1038/nrn2762 20046194

[33] Feld GB Wilhem I Benedict C Rudel B Klameth C Born J. Central nervous insulin signaling in sleep-associated memory formation and neuroendocrine regulation. Neuropsychopharmacology (2016) 41:1540–50. 10.1038/npp.2015.312 26448203 PMC4832015

[34] Santiago JC Hallschmid M. Central nervous insulin administration before nocturnal sleep decreases breakfast intake in healthy young and elderly subjects. Front Neurosci. (2017) 11:54. 10.3389/fnins.2017.00054 28228715 PMC5296307

[35] Pudel V Westenhöfer J Fragebogen zum Essverhalten: Handanweisung. Göttingen: Hogrefe (1989).

[36] Greenwood CE Kaplan RJ Hebblethwaite S Jenkins DJ. Carbohydrate-induced memory impairment in adults with type 2 diabetes. Diabetes Care (2003) 26:1961–6. 10.2337/diacare.26.7.1961 12832296

[37] Plihal W Born J. Effects of early and late nocturnal sleep on priming and spatial memory. Psychophysiology (1999) 36:571–82. 10.1111/1469-8986.3650571 10442025

[38] Janke W Debus G Die Eigenschaftswörterliste (EWL). Göttingen: Hogrefe (1978).

[39] ter Maaten JC Bakker SJ Serne EH ter Wee PM Donker AJ Gans RO. Insulin's acute effects on glomerular filtration rate correlate with insulin sensitivity whereas insulin's acute effects on proximal tubular sodium reabsorption correlation with salt sensitivity in normal subjects. Nephrol Dial Transplant. (1999) 14:2357–63. 10528658 10.1093/ndt/14.10.2357

[40] Koch L Wunderlich FT Seibler J Konner AC Hampel B Irlenbusch S. Central insulin action regulates peripheral glucose and fat metabolism in mice. J Clin Invest. (2008) 118:2132–47. 10.1172/JCI31073 18451994 PMC2350427

[41] Jessen L Clegg DJ Bouman SD. Evaluation of the lack of anorectic effect of intracerebroventricular insulin in rats. Am J Physiol Regul Integr Comp Physiol. (2010) 298:R43–50. 10.1152/ajpregu.90736.2008 19864335

[42] Manin M Balage M Larue-Achagiotis C Grizard J. Chronic intracerebroventricular infusion of insulin failed to alter brain insulin-binding sites, food intake, and body weight. J Neurochem. (1988) 51:1689–95. 10.1111/j.1471-4159.1988.tb01146.x 3053993

[43] Hallschmid M Benedict C Schultes B Born J Kern W. Obese men respond to cognitive but not to catabolic brain insulin signaling. Int J Obes (Lond). (2008) 32:275–82. 10.1038/sj.ijo.0803722 17848936

[44] de Kloet ER Meijer OC de Nicola AF de Rijk RH Joëls M. Importance of the brain corticosteroid receptor balance in metaplasticity, cognitive performance and neuro-inflammation. Front Neuroendocrinol. (2018) 49:124–45. 10.1016/j.yfrne.2018.02.003 29428549

[45] Jacobson L Sapolsky R. The role of the hippocampus in feedback regulation of the hypothalamic–pituitary–adrenocortical axis. Endocr Rev. (1991) 12:118–34. 10.1210/edrv-12-2-118 2070776

[46] Thienel M Wilhelm I Benedict C Born J Hallschmid M. Intranasal insulin decreases circulating cortisol concentrations during early sleep in elderly humans. Neurobiol Aging (2017) 54:170–4. 10.1016/j.neurobiolaging.2017.03.006 28385552

[47] Benedict C Dodt C Hallschmid M Lepiorz M Fehm HL Born J Immediate but not long-term intranasal administration of insulin raises blood pressure in human beings. Metabolism (2005) 54:1356–61. 10.1016/j.metabol.2005.04.026 16154436

[48] Feld GB Diekelmann S. Sleep smart-optimizing sleep for declarative learning and memory. Front Psychol. (2015) 6:622. 10.3389/fpsyg.2015.00622 26029150 PMC4428077

[49] Grillo CA Piroli GG Lawrence RC Wrighten SA Green AJ Wilson SP. Hippocampal insulin resistance impairs spatial learning and synaptic plasticity. Diabetes (2015) 64:3927–36. 10.2337/db15-0596 26216852 PMC4613975

[50] Tschritter O Preissl H Hennige AM Stumvoll M Porubska K Frost R. The cerebrocortical response to hyperinsulinemia is reduced in overweight humans: a magnetoencephalographic study. Proc Natl Acad Sci USA. (2006) 103:12103–8. 10.1073/pnas.0604404103 16877540 PMC1567704

[51] Kullmann S Heni M Veit R Scheffler K Machann J Häring HU. Selective insulin resistance in homeostatic and cognitive control brain areas in overweight and obese adults. Diabetes Care (2015) 38:1044–50. 10.2337/dc14-2319 25795413

[52] Reger MA Watson GS Frey WH II Baker LD Cholerton B Keeling ML. Effects of intranasal insulin on cognition in memory-impaired older adults: modulation by APOE genotype. Neurobiol Aging (2006) 27:451–8. 10.1016/j.neurobiolaging.2005.03.016 15964100

[53] Ott V Benedict C Schultes B Born J Hallschmid M. Intranasal administration of insulin to the brain impacts cognitive function and peripheral metabolism. Diabetes Obes Metab. (2012) 14:214–21. 10.1111/j.1463-1326.2011.01490.x 21883804

[54] Craft S Claxton A Baker LD Hanson AJ Cholerton B Trittschuh EH. Effects of regular and long-acting insulin on cognition and Alzheimer's disease biomarkers: a pilot clinical trial. J Alz Dis. (2017) 57:1325–34. 10.3233/JAD-161256 28372335 PMC5409050

